# A First‐In‐Class Antibody Enabling Detection of Altered Peripheral Non‐Phosphorylated Clusterin With Translational Potential in Dementia

**DOI:** 10.1096/fj.202601699RR

**Published:** 2026-07-14

**Authors:** Hejie Li, Yu Li, Wenping Liang, Liyong Wu, Chao Han, Qianxu Ren, Xi Liu, Yanning Cai, Zhe Wang

**Affiliations:** ^1^ The National Clinical Research Center for Geriatric Disease, Department of Neurology, Advanced Innovation Center for Human Brain Protection Xuanwu Hospital, Capital Medical University Beijing China; ^2^ Department of Neurology Xuanwu Hospital, Capital Medical University Beijing China; ^3^ Department of Neurology The First Affiliated Hospital of Zhengzhou University Zhengzhou China; ^4^ Department of Clinical Biobank Xuanwu Hospital, Capital Medical University Beijing China

**Keywords:** brain‐to‐serum, CLU, dementia, phosphorylation, serum

## Abstract

Clinical diagnosis of dementia, with Alzheimer's disease (AD) as the major form, relies heavily on memory tests and the caregiver descriptions, both of which are subjective. The discovery of reliable biomarkers may facilitate objective diagnosis. The protein clusterin (CLU), encoded by a well‐established AD risk gene, is consistently elevated in AD patients, but its biomarker utility is limited by high interindividual variability. As CLU is produced in most organs and tissues, quantifying CLU secreted specifically from the brain into the bloodstream may help the development of new diagnostic methods. CLU in blood can be phosphorylated at T393‐S394 and/or S396, but these phosphorylations are absent in brain parenchyma. Peripherally administered non‐phosphorylated CLU has been shown to reduce neuroinflammation and AD pathology in mouse models. A monoclonal antibody, 3D3F10, targeting non‐phosphorylated CLU at T393‐S394 or S396 was generated. This antibody showed an ability to distinguish serum samples of dementia and non‐dementia (*p* < 0.001, AUC = 0.897, sensitivity: 81.8%, specificity: 95.5%, Youden index: 0.77), but did not distinguish Parkinson's disease. The direction of the change contradicted our initial hypothesis, and further analyses suggested that phospho‐CLU in dementia patients is unlikely to originate from the brain. These results established 3D3F10 as a novel tool for modification‐specific CLU detection, indicated a potential of non‐phospho‐CLU as a biomarker for dementia, and peripheral phospho‐CLU might play a role in pathogenesis.

## Graphical abstract

Non‐phosphorylated clusterin is decreased in the serum of dementia patients but not in Parkinson's disease, and phosphorylated clusterin in serum does not originate from the brain. 3D3F10, a monoclonal antibody specific to non‐phosphorylated clusterin, enables modification‐specific detection with diagnostic potential for dementia.

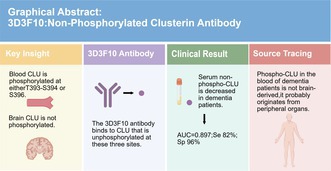

## 1 Introduction

Dementia, with Alzheimer's disease (AD) as the predominant form in aged adults, affects tens of millions worldwide [1, 2]. In AD, extracellular amyloid‐β (Aβ) aggregates and hyperphosphorylated tau in neurons constitute hallmark pathological features [3, 4, 5, 6, 7]. Aβ and phospho‐tau, as well as a number of biomarkers originated in the brain, can enter the circulation via the blood–brain barrier or glymphatic system [8, 9]; however, their extremely low blood concentrations necessitate highly sensitive detection methods, which limits practical applications [10].

Clusterin (CLU), encoded by a well‐established AD risk gene, has been implicated in Aβ clearance and transport from the brain to the blood [11] [12, 13]. Functional studies showed that CLU can both prevent Aβ oligomerization and promote its clearance through glial‐mediated phagocytosis [12, 13, 14]. CLU also facilitates Aβ excretion from the brain into peripheral circulation, and its interaction with APP and Aβ can modulate neuritic plaque formation [15]. CLU is upregulated in the brain [16, 17, 18, 19, 20], cerebrospinal fluid (CSF) [21, 22, 23], and plasma [19, 24, 25, 26, 27] in AD, but blood levels vary substantially among individuals, possibly because of ubiquitous expression in multiple organs and tissues [11, 28]. This high variability reduces the reliability of total serum CLU as a biomarker for AD [29].

Recent mass spectrometry studies detected phosphorylated CLU at Thr393–Ser394 or Ser396 in blood, but these phosphorylations are absent in the brain parenchyma [30]. This differential post‐translational modification pattern led us to hypothesize that brain‐derived non‐phosphorylated CLU may accumulate in peripheral blood in AD or dementia and could serve as a more specific biomarker than total CLU. Moreover, the peripheral administration of non‐phosphorylated CLU in mouse models has been shown to reduce AD pathologies and neuroinflammation [31], although the exogenous CLU could not enter brain parenchyma, which suggests therapeutic potential.

However, there is currently no tool to specifically detect non‐phosphorylated CLU in blood except mass spectrometry, limiting investigation of its clinical and mechanistic significance. To address this gap, we generated a monoclonal antibody, 3D3F10, specifically recognizing CLU without phosphorylation at Thr393, Ser394, or Ser396. This first‐in‐class reagent enables the detection of non‐phosphorylated CLU in serum and provides a framework to explore its translational potential and mechanistic investigation in dementia.

## 2 Methods and Materials

### 2.1 Antigens for Monoclonal Antibody Generation

Non‐phosphorylated antigen‐1 (HTSDSDVPSGC) and antigen‐2 (TVASHTSDSD) were conjugated to keyhole limpet hemocyanin (KLH) and mixed to immunize Balb/c mice.

### 2.2 Preparation of Hybridoma Cells

BALB/C mice provided by the Beijing Laboratory Animal Research Center were used as the immunized animals, and the first subcutaneous multipoint injection was performed using the mixed immunogen (emulsified with 0.1 mL complete Freund's adjuvant) as the immunogen at a dose of 0.1 mL/mouse. After 1 month, immunization was strengthened by two more injections. To extract serum from these mice, the mice were held in a restrainer. The tails were wiped with warm water (~40°C) or an alcohol cotton Q‐tip to dilate the blood vessels. A small horizontal cut at the tip of the tail was quickly made with a sharp sterile surgical blade (avoid longitudinal cutting to prevent injury to the tail vertebra). Blood was collected with a capillary tube. After blood collection, a dry cotton ball was pressed to the cutting site to stop bleeding. The serum antibody potency was determined by indirect competition ELISA. Hybridoma cells were screened by limited dilution or soft agar plate method. To collect splenocytes, the mice were euthanized by isoflurane inhalation (5% isoflurane ventilated with air containing 80% oxygen), and death of the mice was confirmed by observing the movement of chest and abdomen that indicate breath. Splenocyte cells taken from the mice were fused with myeloma cells SP2/0 at a ratio of 4:1. The hybridoma cells were screened by limited dilution or soft agar plate method, and the monoclonal hybridoma cell line which stably secreted the monoclonal antibody against human non‐phosphorylated CLU protein was obtained and named as 3D3F10 mouse hybridoma cell line. The 3D3F10 mouse hybridoma cell line was frozen and preserved. Antibodies in conditioned media of the hybridoma were purified using protein A/G beads.

### 2.3 Cell Storage

When the hybridoma density in the expanded culture reaches 90%, the supernatant was discarded, and the flask was gently tapped at both sides. Cells were resuspended and centrifuged at 1000 rpm for 5 min. Cell pellets were resuspended with a cryopreservation solution composed of 70% FBS (Procell, Wuhan, China), 20% DMEM medium (Servicebio, Wuhan, China) which includes 10% FBS, and 10% DMSO (Sigma‐Aldrich, Shanghai, China), then transferred to the programmed cooling box, left overnight in −80°C freezer, and transferred to liquid nitrogen for long‐term storage the next day.

### 2.4 Antibody Purification

The 3D3F10 monoclonal cell line (3D3F10 mouse hybridoma cell line) was selected for serum‐free cell expression to obtain 3D3F10 cell expression supernatant. For antibody purification, the cell culture supernatants were processed slowly through a protein‐A agarose column. After the supernatant flowed through the column, the binding antibody was eluted with glycine elution buffer (pH 2.5), and the desired purified antibody was obtained. The eluate was immediately dialyzed in PBS (Solarbio, Beijing, China) at 4°C overnight. The concentration of purified antibody was determined by BCA measurement. The purified antibody was frozen at −80°C for future use.

### 2.5 Characterization of Antibody Purity

The purified antibody was subjected to SDS‐PAGE electrophoresis and stained with Coomassie Brilliant Blue R250 (Solarbio, Beijing, China) to examine the purity and integrity of the antibody.

### 2.6 Antibody Identification

The potency of the 3D3F10 monoclonal antibody was assessed by ELISA, and the antibody concentration was measured with a BCA protein concentration assay kit.

For the purified antibody ELISA potency assay, proteins antigen‐1: HT^393^S^394^DS^396^DVPSG and ag2: TVASHT^393^S^394^DS^396^D, p‐ag1: TVASHT393(p)S394DS396D, p‐ag2: TVASHT393S394(p)DS396D, and p‐ag3: TVASHT393S394DS396(p)D were diluted to 0.1 μg/mL in a PBS coating solution (pH 7.4) and added to the coated plates, which also included a negative control group that was not coated. A volume of 100 μL was dispensed into each well, with each group being tested in triplicate. The plates were then refrigerated overnight at 4°C.

Following coating, the plates were washed three times with PBS solution. Next, 200 μL of sealing solution was added to each well and incubated at 37°C for 1 h. Then, 100 μL of diluted 3D3F10 monoclonal antibody was added to each well and incubated at 37°C for 1 h. The liquid was then discarded, and the plates were washed three times with PBS coating solution. Following this, 100 μL of diluted secondary antibody (goat anti‐mouse‐HRP, Beyotime, Shanghai, China) diluted in PBS coating solution at a ratio of 1:20 000 was added to each well and incubated at 37°C for 1 h. The plate was washed four times with PBS coating solution, and then 100 μL of TMB color developing solution was added to each well, followed by a 15‐min incubation at 37°C. To halt the reaction, 100 μL of 1 M HCl solution was added to each well. The absorbance was then measured at 450 nm using a microplatereader (Potenov, Beijing, China). The dilution corresponding to the well with an optical density (OD) value exceeding 2.1 times the OD value of the negative control is considered the potency of the sample.

### 2.7 Dementia Patients

Mixed dementia patients were recruited from Xuanwu Hospital. Diagnoses were made by two physicians using the DSM‐5 criteria [32]. Detailed etiological subtyping (AD, vascular, etc.) was not available for this cohort.

### 2.8 Patients With Parkinson's Disease

The patients were recruited from the Department of Neurology at Xuanwu Hospital. The diagnosis of Parkinson's disease (PD) complies with the guidelines of the Movement Disorder Society [33]. Patients had a Hoehn–Yahr stage ≤ 3, and disease duration was ≤ 5 years. All PD patients' diagnosis was confirmed by two independent senior neurology physicians, and were from in‐patients. PD patients with dementia were excluded from this study.

### 2.9 Mouse Brain Stereotactic Injection

Wild‐type C57BL/6J mice (RRID: IMSR_JAX:000664) and 5xFAD AD model mice (RRID: MMRRC_034848‐JAX) were purchased from Cyagen Biosciences Inc. (Suzhou, Jiangsu, China). The ARRIVE guidelines were followed throughout the study. All efforts were made to minimize the number of animals used and ensure minimal suffering. The mice were maintained in a specific pathogen‐free (SPF) environment with a 12‐h day‐night cycle, a controlled temperature of 25°C, and a relative humidity of 45%–55%. All mice were used at the indicated ages, and the specific numbers of mice used in each experiment are detailed in the figure legends.

Mice were anesthetized by haloflurane (3%) inhalation. After anesthesia, the mice were fixed on a brain stereotaxic coordinator (Biowill, ShangHai, China). The bregma point was taken as the origin of the three‐dimensional coordinates, and the viruses were injected into the lateral ventricles at a rate of 0.2 μL/min. After the injection, the needle was left in the mouse brain for 10 min, then the needle was slowly withdrawn, and the wound was sterilized and closed with a medical suture. Rectal temperature was maintained at 37.0°C ± 0.5°C throughout the experimental and recovery periods. The incision was coated with lidocaine gel for 3 days for analgesia. The mice were sacrificed 3 weeks post stereotactic injection.

To collect blood and brains, the mice were euthanized by deep isoflurane inhalation (5% isoflurane ventilated with air containing 80% oxygen), and death of the mice was confirmed by observing the movement of the chest and abdomen that indicate breath. The mice were decapitated to collect blood or brains.

### 2.10 Plasmid Construction

The wild type human CLU cDNA (coding for NP_001822.3) was used as a template for site‐directed mutagenesis using fusion PCR (Bio‐Rad, Shanghai, China). The PCR program consisted of 30 cycles, including denaturation at 95°C for 15 s, annealing at 65°C for 30 s, and elongation at 72°C for 30 s. Mutant cDNAs were digested using HindIII and XbaI restriction enzymes and were inserted into the pcDNA4‐mic‐hisA vector between HindIII and XbaI. The plasmid sequences were verified by Sanger sequencing.

### 2.11 Cell Culture and Transfection

HEK293 cells purchased from American Type Culture Collection (CRL‐1573; ATCC, Manassas, VA, USA) were cultured in high‐glucose Dulbecco's Modified Eagle Medium (Servicebio, Wuhan, China) containing 10% fetal bovine serum (ProCell Therapies, New York, NY, USA), 1 mM sodium pyruvate, 4 mM L‐glutamine, and maintained at 37°C in an incubator containing 5% CO_2_. Authentication of cell lines was conducted by Procell (Procell, Hubei, China). We performed mycoplasma testing every 20 days using the MycoBlue Mycoplasma Detector (Vazyme, Nanjing, China). Plasmids were transfected into cells with Lipo8000 Transfection Reagent (Beyotime, Shanghai, China). Before transfection (18–24 h prior), the cells were inoculated into six‐well plates with approximately 200 000 to 700 000 cells per well to achieve a cell density of approximately 70%–80% the following day. 125 μL of antibiotic and serum‐free DMEM culture medium were added to each well designated for transfection. Next, 1 μg of plasmid DNA was added and mixed thoroughly by gently pipetting. Then, 4 μL of Lipo8000 Transfection Reagent was introduced and mixed again by gently pipetting. The plates were placed in the incubator, and after 4 h, the culture medium was replaced with 1 mL of fresh complete culture medium containing serum and antibiotics.

### 2.12 Immunoprecipitation

The 3D3F10 antibody (1 μg) was incubated with protein A/B beads (Smart‐Lifesciences, Jiangsu, China) and blocked with 1% BSA in RIPA buffer (Solarbio, Beijing, China) for 1 h. The cell culture medium obtained after plasmid transfection, as well as cerebrospinal fluid and serum samples—with or without dephosphorylation—were diluted 1:300 in RIPA buffer. Samples were then reacted for 1 h with magnetic beads that did not have an antibody conjugate, followed by centrifugation to remove insoluble protein aggregates, and then added to 3D3F10 antibody. The reactions were incubated overnight at 4°C with vertical rotation (Kylin‐Bell, Jiangsu, China). The proteins bound to the beads were eluted using 2× SDS sample buffer for Western blot analysis.

### 2.13 Dephosphorylated and Undephosphorylated Processing of Serum

To prepare dephosphorylated serum, 1 μL of human serum was mixed with 39 μL of deionized water, followed by the addition of 5 μL of MnCl_2_ (B1761s; NEB, Beijing, China) solution, 5 μL of PMP Buffer (B0761S; NEB, Beijing, China), and 1 μL of Lambda Protein Phosphatase (P0753s; NEB, Beijing, China). This mixture was incubated for 1 h at 30°C. For the undephosphorylated serum, the same procedure was followed, substituting Lambda phosphatase with 5 μL of Protease and Phosphatase Inhibitor Cocktail (HY‐K0013; MCE, Shanghai, China).

### 2.14 ELISA

The 3D3F10 antibody was coated onto 96‐well plates as the capture antibody for the sandwich ELISA assay. Whole serum samples were diluted 1:250 with PBS, and 100 μL of the diluted serum was added to the plates. After incubating overnight at 4°C, detection was performed using an ELISA Kit for human Clusterin (SEB180Hu; Cloud‐Clone Corp., Wuhan, China). The enzyme‐substrate reaction was terminated by sulfuric acid, and the color change was measured spectrophotometrically at a wavelength of 450 ± 10 nm (Potenov, Beijing, China). The concentration of clusterin in the sample was determined by comparing the optical density (O.D.) of the samples to a standard curve.

### 2.15 Western Blot

Proteins were resolved by sodium dodecyl sulfate (SDS) polyacrylamide gel electrophoresis and electro‐transferred to nitrocellulose membranes. The membranes were blocked in phosphate‐buffered saline containing 5% BSA for at least 1 h. Anti‐Clusterin antibodies (PAB180Hu01; Cloud‐Clone Corp for the detection of CLU mutants as the epitope of this antibody is the entire α‐chain of human CLU and D4B6P; Cell Signaling Technology, Shanghai, China) were used to detect Clusterin. Anti‐phosphorylated serine and threonine antibody was from Santa Cruz Biotechnology (sc‐81 514, Santa Cruz, CA, USA).

### 2.16 Statistical Analyses

Statistical analyses were performed using GraphPad Prism 8 (GraphPad Software, San Diego, CA, USA). Data are presented as mean ± standard deviation (SD) for normally distributed variables or median (interquartile range, IQR) for non‐normally distributed variables. Normality was assessed using the Shapiro–Wilk test. Comparisons between two groups were performed using paired or unpaired two‐tailed Student's *t*‐test as appropriate. Comparisons among multiple groups were conducted using one‐way ANOVA followed by Tukey's post hoc test. Receiver operating characteristic (ROC) curve analysis was performed to evaluate the diagnostic performance of 3D3F10, and the area under the curve (AUC) with 95% confidence intervals (CIs) was reported. The optimal cutoff value was determined using the Youden index. Effect sizes and 95% CIs were calculated where applicable. A two‐sided *p*‐value < 0.05 was considered statistically significant. *n* values represent the numbers of biological replications for biochemical experiments. The graphical abstract was created with BioGDP.com [34].

## 3 Results

### 3.1 Brain‐Derived CLU Enters the Circulation and Alters Serum CLU Homeostasis

**FIGURE 1:**
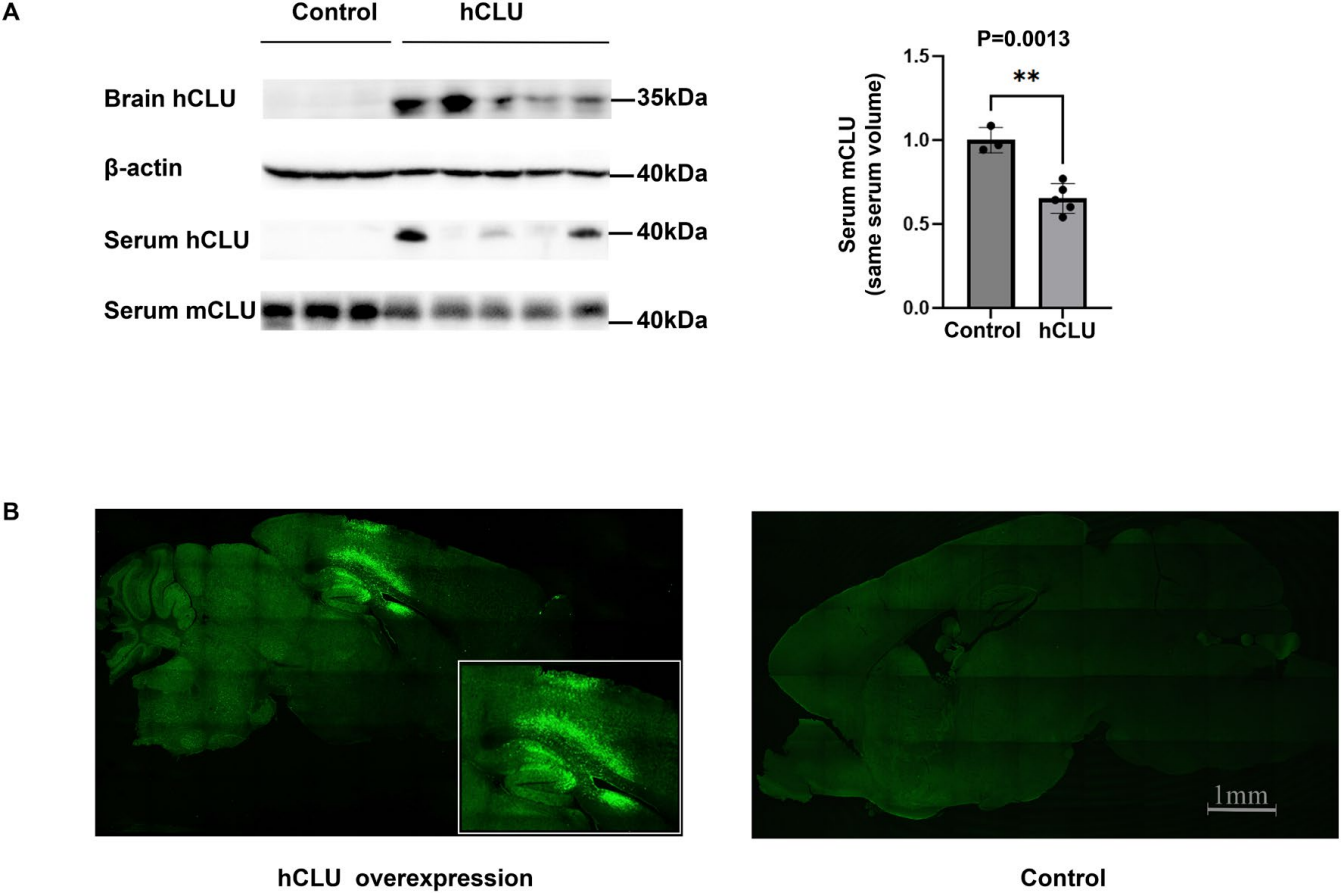
Brain‐derived CLU entered the bloodstream and significantly reduced serum CLU levels in mice. (A) AAV9 expressing hCLU was injected into wild‐type C57BL/6 mice. Two weeks later, hCLU levels in the brain and serum, as well as endogenous mouse CLU (mCLU) levels, were quantified using Western blot analysis. Control AAV: *n* = 3, CLU overexpression: *n* = 5. Data are expressed as mean ± SD. **p < 0.01 by unpaired student's *t*‐test. (B) Mouse brain slice with overexpressed hCLU was stained with anti‐FLAG antibody to indicate brain areas expressing hCLU.

To determine whether increased brain CLU expression affects serum CLU levels, we injected 2‐month‐old wild‐type mice intracerebroventricularly (ICV) with an AAV9 virus expressing FLAG‐tagged human CLU (hCLU). An AAV9 expressing FLAG tag served as control. The expression of hCLU was driven by the ubiquitous CMV promoter. Two weeks after virus injection, hCLU expressed in the brain was readily detectable in both brains and sera. Notably, endogenous mouse CLU (mCLU) in the sera of these mice was significantly reduced as compared to mCLU in the sera of mice expressing FLAG tag in the brain (Figure 1A) (the anti‐hCLU and anti‐mCLU antibodies are specific for hCLU and mCLU, respectively), which suggested a homeostatic regulation of blood CLU level: at least in this experimental setting, when brain‐produced CLU enters the circulation, peripheral CLU production is decreased either by lowered synthesis or faster turnover. This may result in a higher portion of brain‐derived CLU in the blood. Immunostaining confirmed that hCLU was highly expressed in the peri‐ventricle area, and was also detectable in cells distal to ventricles.

### 3.2 Generation, Validation, and Structural Basis of 3D3F10 Monoclonal Antibody Specific for Non‐Phosphorylated CLU

**FIGURE 2:**
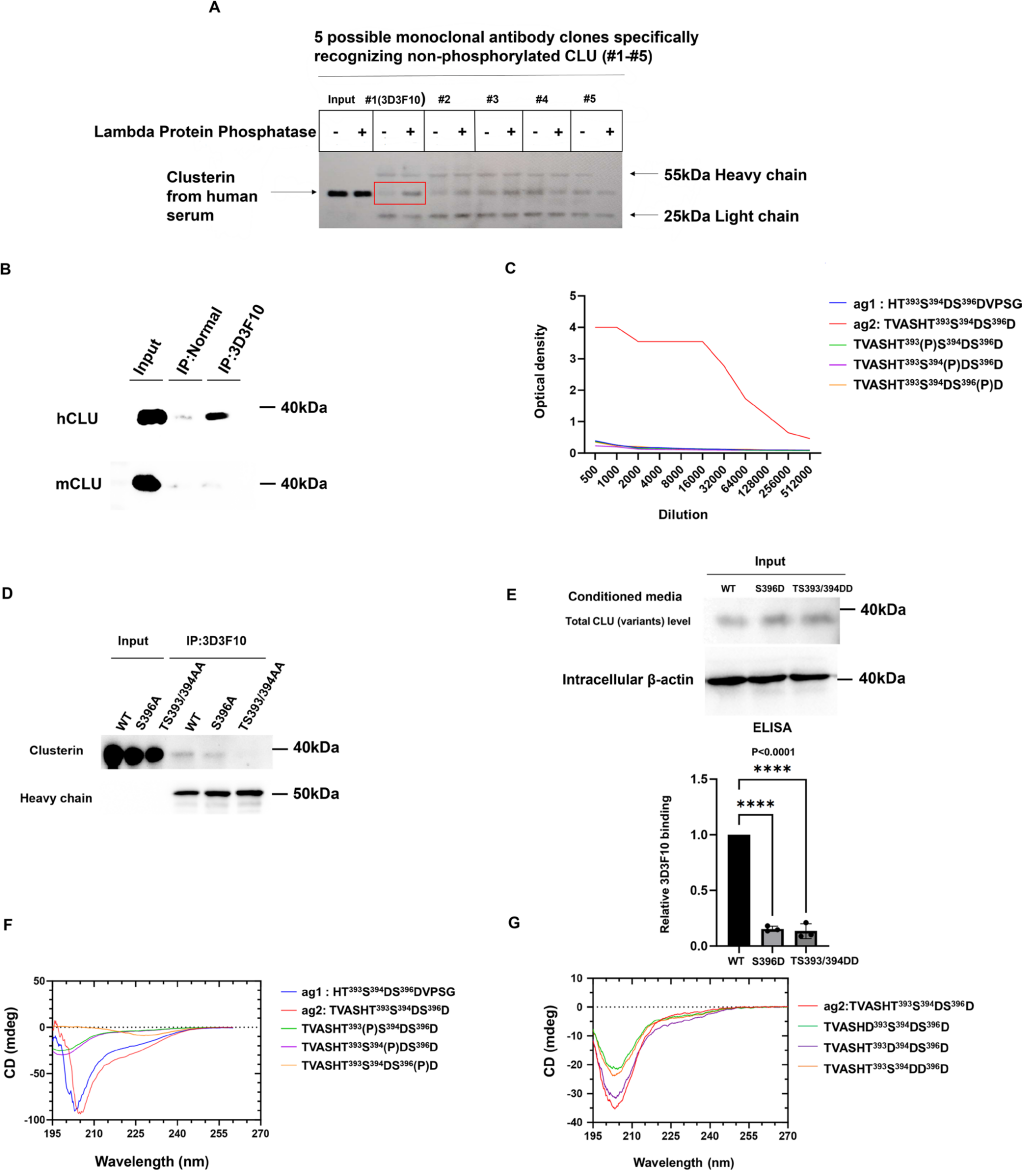
The 3D3F10 monoclonal antibody specifically reacted with hCLU in the absence of phosphorylation at T^393^, S^394^, and S^396^. (A) Human serum was treated with or without λ‐protein phosphatase and then immunoprecipitated using antibodies from five hybridoma cell lines. (B) The sera of human and C57BL/6 wild‐type mice were subjected to immunoprecipitation with 3D3F10. The precipitated proteins were analyzed for hCLU and mCLU levels. *n* = 3 independent repeats. (C) Antigens and phosphorylated antigens were coated onto plates, and 3D3F10, in a series of dilutions, was incubated with these antigens to determine binding specificity and affinity. (D) Wild‐type hCLU, the hCLU S396A mutant and the hCLU T393A/S394A double mutant were overexpressed in HEK293 cells, and CLU variants in the conditioned media were immunoprecipitated using the 3D3F10 antibody and analyzed by Western blotting with an anti‐hCLU antibody (Cloud‐Clone). *n* = 4 independent repeats. (E) phospho‐mimicking mutants of CLU, CLU_T393D/S394D_ and CLU_S396D_, as well as wild type CLU were overexpressed in HEK293 cells and CLU variants in conditioned media were quantified by ELISA using 3D3F10 antibody. The values were expressed as the ratios to CLU_WT_. Equal expressions of CLU variants in conditioned media were determined by Western blot using a total CLU antibody. *n* = 3 independent repeats. ****p < 0.0001 by one‐way ANOVA followed by Tukey's post hoc test. (F) The structures of antigen‐1, antigen‐2, and phosphorylated antigen‐2 at T393, S394 and S396 were analyzed by circular dichroism. (G) The structures of antigen‐2, and phospho‐mimicking mutants of antigen‐2 were analyzed by circular dichroism.

Thr^393^‐Ser^394^ and Ser^396^ phosphorylated CLU was detected in the blood but not in the brain. Therefore, we reasoned that if in AD or dementia patients, the brains produce higher levels of CLU, there would be more brain‐derived non‐phospho‐CLU in the blood. As a consequence, phosphorylated CLU generated in the periphery would decrease with total peripheral CLU, resulting in lower phospho‐CLU and higher non‐phospho‐CLU in the blood. To test this hypothesis, two overlapping peptides from hCLU containing non‐phosphorylated Thr^393^–Ser^394^ and Ser^396^ (ag1 (antigen‐1): HT^393^S^394^DS^396^DVPSG and ag2: TVASHT^393^S^394^DS^396^D) were combined to immunize mice, and antibodies from 15 hybridoma clones showed relatively high affinity with the antigens. To examine the binding of these antibodies to hCLU in human sera and to screen antibodies with low or no binding with phosphorylated hCLU, we first tested immunoprecipitation of hCLU from human sera treated with or without λ‐protein phosphatase that removes phosphate from proteins. All the antibodies could precipitate hCLU from the serum, but only the antibody from clone 3D3F10 displayed significantly enhanced immunoprecipitation upon λ‐protein phosphatase treatment (Figure 2A). Immunoprecipitation suggested that 3D3F10 interacted with hCLU in human sera, but not with mCLU in mouse sera (Figure 2B).

To further confirm the specific binding of antibody 3D3F10 to non‐phosphorylated antigens, we performed a titration of this antibody. Equal amounts of the two antigens and three phosphorylated antigens (p‐ag1: TVASHT^393^(p)S^394^DS^396^D, p‐ag2: TVASHT^393^S^394^(p)DS^396^D, and p‐ag3: TVASHT^393^S^394^DS^396^(p)D, all based on ag2) were coated onto 96‐well plates, and 3D3F10 antibody (0.56 mg/mL) was serially diluted. ELISA results indicated that the binding of 3D3F10 to antigen‐2 was over 10 times higher than to antigen‐1 and phosphorylated antigens even at the dilution up to 1:128 000 (~4.5 ng/mL) (Table 1 and Figure 2C). Hence, the non‐phosphorylated T^393^, S^394^, S^396^, and the residues TVASH N‐terminally flanking these phosphorylation sites are crucial for 3D3F10 binding.

**TABLE 1:** Binding affinities of 3D3F10 with phospho‐ and non‐phospho‐antigens.

Dilution	3D3F10 monoclonal antibody				
	ag‐1	ag‐2	ag‐2393P	ag2394P	ag2396P
0	0.121	0.124	0.094	0.068	0.090
500	0.392	4.000	0.369	0.234	0.350
1000	0.258	4.000	0.260	0.203	0.228
2000	0.182	3.549	0.156	0.132	0.213
4000	0.178	3.549	0.154	0.117	0.162
8000	0.151	3.549	0.149	0.109	0.136
16 000	0.130	3.549	0.140	0.096	0.133
32 000	0.127	2.771	0.121	0.095	0.129
64 000	0.105	1.730	0.091	0.091	0.127
128 000	0.101	1.203	0.087	0.088	0.106
256 000	0.100	0.653	0.081	0.085	0.105
512 000	0.099	0.460	0.078	0.083	0.104
Blank	0.093	0.111	0.075	0.081	0.102
	> 1000	> 512 000	> 1000	1000	1000

To confirm that 3D3F10 binds to the designed epitopes of hCLU, we generated two hCLU mutants (T393A‐S394A and S396A) and overexpressed the wild‐type hCLU and the two mutants in HEK293 cells. The conditioned media of the cells were subjected to 3D3F10 immunoprecipitation. At similar levels, the S396A mutant only weakly bound to 3D3F10 as compared to hCLU_WT_, whereas the T393A–S394A double mutant abolished the immunoprecipitation (Figure 2D). Similarly, ELISA using 3D3F10 indicated that the phospho‐mimicking mutants CLU_T393D/S394D_ and CLU_S396D_ overexpressed in HEK293 and secreted into conditioned media displayed extremely weak binding with 3D3F10 as compared with wild type CLU (Figure 2E). As such, all the three phosphorylation sites are required for the maximum binding of 3D3F10, and T^393^ and S^394^ are crucial for the binding.

We also tested 3D3F10 for Western blot of CLU in human serum, but the antibody produced no convincing protein band. This led us to speculate that phosphorylation of CLU at the three sites may prevent the binding with 3D3F10 through a structural effect. When the structure required for 3D3F10 binding is disrupted by phosphorylation or by SDS denaturation during Western blot, 3D3F10 can no longer bind CLU. Circular dichroism (CD) scanning revealed that both antigen‐1 and antigen‐2 are rich in helical structure, and the phosphorylation at any of the three sites in antigen‐2 drastically reduced helix content (Figure 2F). The phospho‐mimicking mutants (T393D, S394D, and S396D) of antigen‐2 reduced helix content as well, but to a much lesser extent compared with the phosphorylated antigen‐2 peptides. (Figure 2G).

Sequencing of clone 3D3F10 revealed that the three complementarity‐determining regions (CDR) of the heavy chain are SYWMH (VHCDR1), DIHPNSGHTTHNEKFKD (VHCDR2), and RMPSTGWFAY (VHCDR3). The CDRs of the light chain are RSSRSLVHSNGNTNLH (VLCDR1), KVSNRLS (VLCDR2), and SQSTH (VLCDR3).

### 3.3 Serum Non‐Phospho‐CLU Distinguishes Dementia but Not Parkinson's Disease From Controls

**FIGURE 3:**
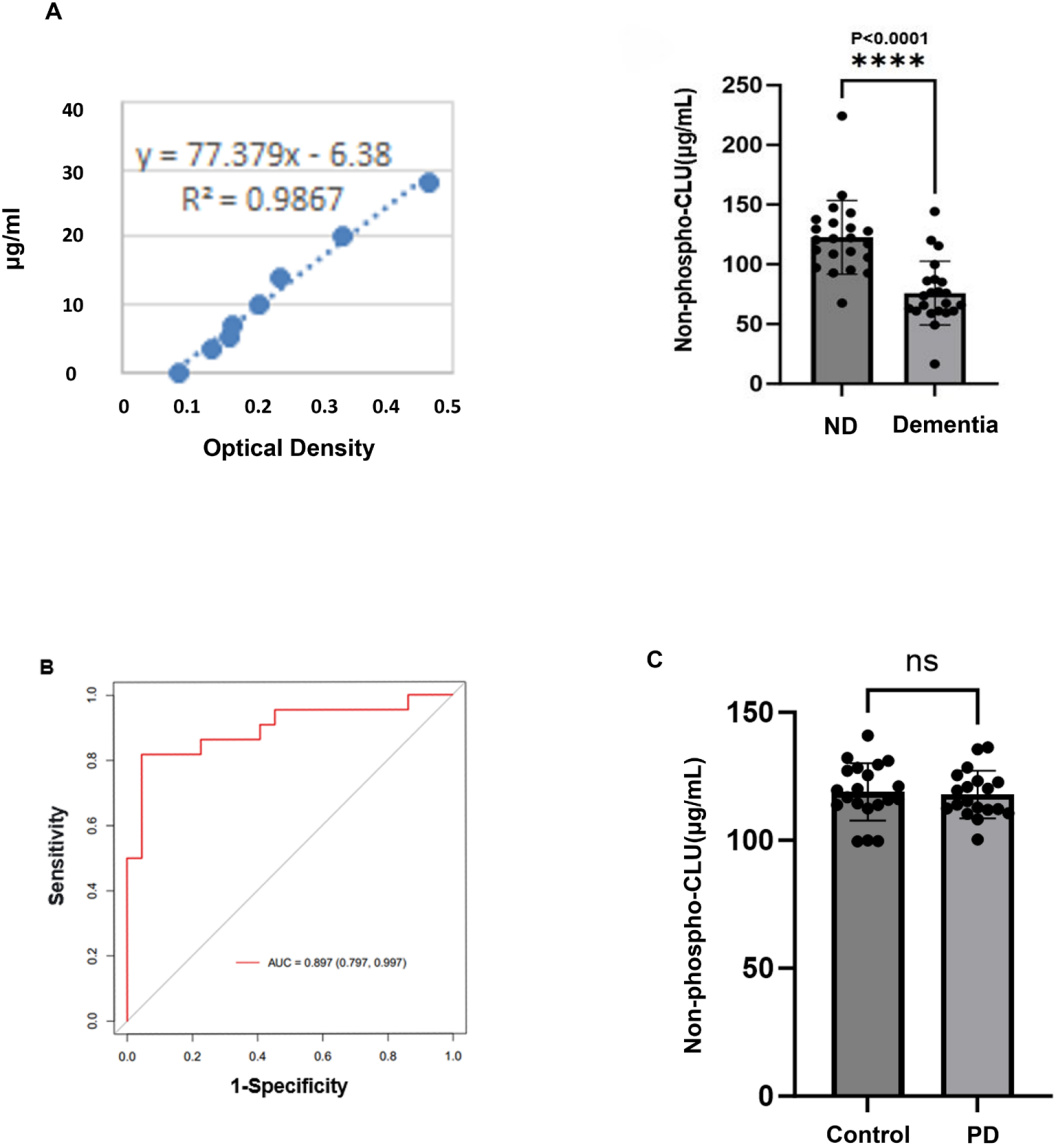
Non‐phospho‐CLU levels were significantly decreased in dementia patients. (A) The concentrations of non‐phosphorylated CLU in the sera of 22 demented individuals and 22 non‐demented controls were measured using ELISA with the 3D3F10 antibody. Our previous study indicated that total CLU levels did not differ between the two groups. Data are expressed as mean ± SD. ****p < 0.0001 by unpaired student's *t*‐test. (B) The diagnostic potential of non‐phosphorylated CLU for distinguishing between clinical states (ND vs. dementia) was assessed using receiver operating characteristic (ROC) curve analysis. (C) The concentrations of non‐phosphorylated CLU in the sera of 20 PD patients and 19 healthy controls were measured using ELISA with the 3D3F10 antibody. Statistics by unpaired student's *t*‐test.

To test if the 3D3F10 antibody could differentiate the serum samples from non‐demented (ND) and demented subjects, non‐phosphorylated CLU levels in the sera of 22 dementia patients and 22 age‐ and sex ‐matched ND subjects (Table S1) were determined by ELISA with 3D3F10 coated onto the plates. We previously reported that there was no statistical difference in total serum CLU level between these two groups of serum samples [15]. The level of non‐phosphorylated CLU in the ND group was 122.6 ± 30.9 μg/mL (mean ± SD), whereas the level decreased to 75.92 ± 26.72 μg/mL in the dementia group (*p* < 0.0001) (Figure 3A and Table S2). Therefore, compared to the ND group, non‐phosphorylated CLU in the dementia group was significantly decreased. Despite the relatively small sample size, ROC curve analysis revealed robust discrimination between dementia and ND subjects, with an AUC of 0.897 (95% CI: 0.797–0.997). Using an optimal cutoff value of 89.977 μg/mL derived from the Youden index, 3D3F10 achieved a sensitivity of 81.8% (95% CI: 63.6%–95.5%) and specificity of 95.5% (95% CI: 86.4%–100%), with an overall diagnostic accuracy of 88.6% (95% CI: 79.5%–97.7%). (Figure 3B). By contrast, non‐phospho‐CLU in the sera of PD (Table S1) was indistinguishable from that in age‐ and sex‐matched control cohort (Figure 3C), suggesting that the change of serum non‐phospho‐CLU is not common for all neurodegenerative diseases.

### 3.4 Brain‐Derived CLU Unlikely Contributes to Serum Phospho‐CLU

Although the ELISA results suggested an efficacy of 3D3F10 in differentiating dementia and ND, the change of serum non‐phosphorylated CLU in dementia was contrary to what we expected. Hence, we reasoned that although phospho‐CLU was not found in brain parenchyma, it might be secreted to the CSF soon after synthesis and phosphorylation. Alternatively, CLU might be phosphorylated extracellularly after being secreted from cells. Moreover, the CLU might become phosphorylated during transcytosis from the brain to the blood in cells within the blood–brain barrier or blood vessels.

To examine if hCLU can be phosphorylated along the secretion pathway or extracellularly, we first analyzed the protein sequence of hCLU. hCLU is a secreted protein through the canonical secretion pathway [28], and therefore exempted from phosphorylation by most kinases that are cytoplasmic. Up to date, the best known protein serine/threonine kinase that phosphorylates secreted proteins in mammals is FAM20c whose substrates contain a S‐x‐E/^P^S motif (x refers to any amino acid and E/^p^S refers to either glutamic acid or phospho‐serine) [35]. While hCLU does not contain the S‐x‐E motif, if S^396^ is phosphorylated first, a S^394^‐x‐^p^S^396^ motif is created. However, how S^396^ can be first phosphorylated is unknown.

**FIGURE 4:**
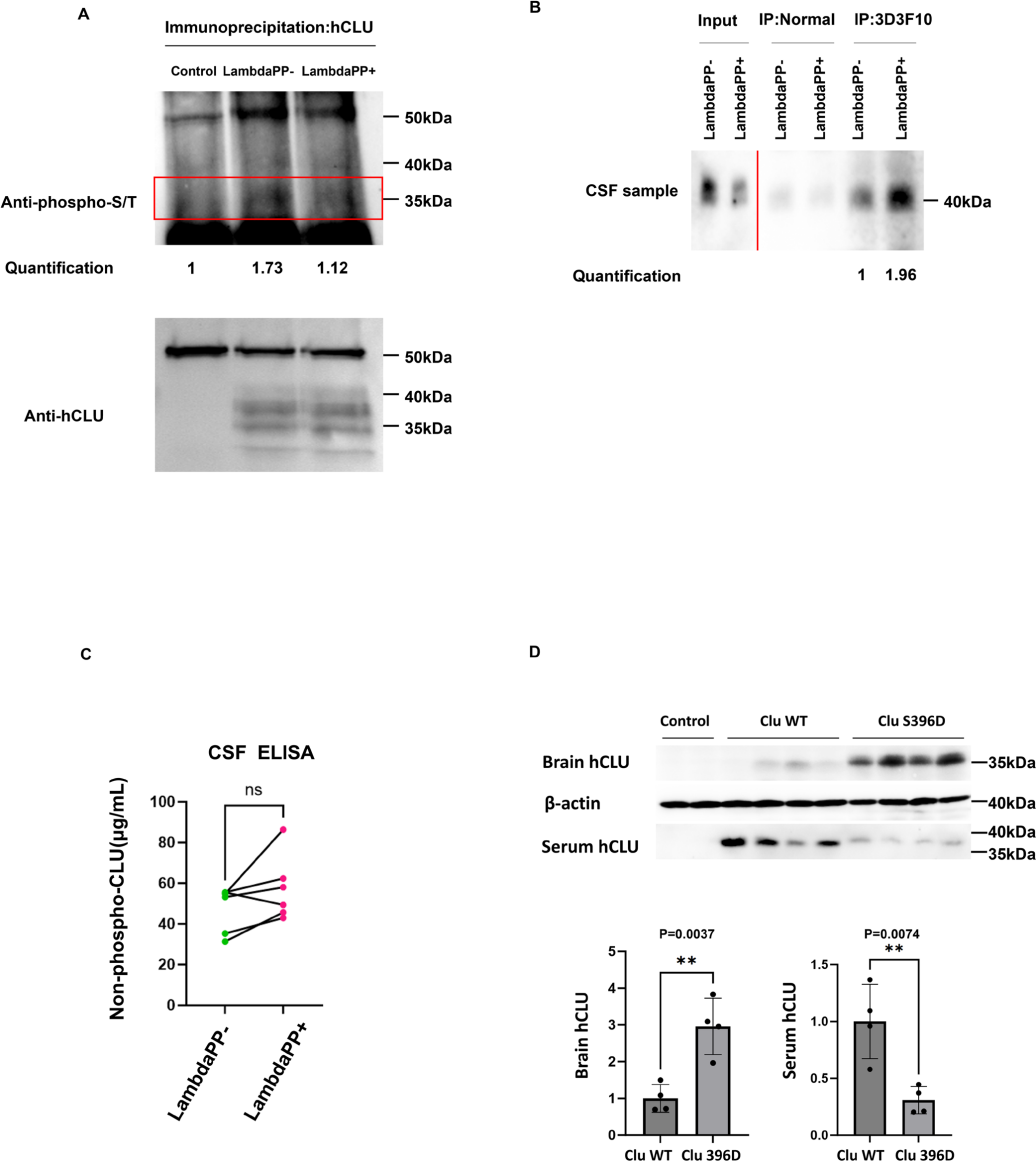
The brain is unlikely to be the source of phosphorylated CLU in serum. (A) Human CSF was treated with (LambdaPP+) or without (LambdaPP−) λ‐protein phosphatase, and hCLU was immunoprecipitated using an anti‐hCLU antibody or mouse normal IgG as control. The precipitates were then blotted for phospho‐serine. CLU protein in CSF is highly and differentially modified (e.g., N‐glycosylation), resulting in multiple protein bands. The red box band indicates phospho‐CLU that was diminished upon de‐phosphorylation. (B) CSF samples from AD patients were treated with (LambdaPP+) or without (LambadPP−) λ‐protein phosphatase, and hCLU was immunoprecipitated using the 3D3F10 antibody or mouse normal IgG (as a control). The precipitates were blotted with an anti‐hCLU antibody. CSF from five ad patients were tested, and this result is from one of the three samples where dephosphorylation slightly increased immunoprecipitation of CLU by 3D3F10. (C) CSF samples from AD patients were treated with (LambdaPP+) or without (LambdaPP−) λ‐protein phosphatase, and the levels of non‐phosphorylated hCLU at the three sites were quantified by ELISA using the 3D3F10 antibody. *n* = 6 CSF samples that were treated with or without λ‐phosphatase. Statistics by paired student's *t*‐test. (D) Wild‐type hCLU and the S396D phospho‐mimicking mutant were overexpressed in mouse brains. hCLU levels in both brain and serum were analyzed by blotting. Control AAV injected mice: *n* = 2, CLUwt overexpressing mice: *n* = 4, CLU_S396D_ mutant overexpressing mice: *n* = 4. Statistics by unpaired student's *t*‐test. Data are expressed as mean ± SD. **p < 0.01.

To investigate the presence of phospho‐CLU in CSF, we performed immunoprecipitation using an anti‐CLU antibody against the C‐terminal half (the α‐chain) of hCLU from human CSF and then Western blotted the precipitates with an anti‐phospho‐serine antibody. CLU isolated from non‐AD CSF was blotted by the anti‐phospho‐serine antibody, and the blots were apparently reduced upon λ‐protein phosphatase treatment of CSF (Figure 4A), supporting the idea that CLU in the brain can be phosphorylated, but this result does not reveal phosphorylation site(s) in CLU. Phosphoproteomics analysis of human CSF using mass spectrometry failed to convincingly identify phosphorylation at T^393^, S^394^, or S^396^. Instead, S^210^ in the N‐terminus of CLU was found to be phosphorylated. However, S^210^ is not within a typical FAM20c target motif, either.

To examine if CSF CLU is phosphorylated at T^393^, S^394^, or S^396^ in AD patients, we collected CSF sample from five ad patients, and performed immunoprecipitation using 3D3F10. The CSFs were pre‐treated with or without λ‐protein phosphatase before immunoprecipitation. In three of the five CSF samples, the immunoprecipitation was slightly enhanced by dephosphorylation (Figure 4B). Consistently, ELISA using the 3D3F10 antibody also revealed in five out of a total of six AD patients (different patients with those in Figure 4B) a minor increase in non‐phosphorylated CLU upon λ‐protein phosphatase treatment (Figure 4C). Therefore, a small portion of CLU in the CSF of AD patients may be phosphorylated at the three sites, but the phosphorylation is not universal among all AD patients. The phospho‐CLU in CSFs of AD patients was not necessarily locally phosphorylated in the brain (see Discussion).

More importantly, when the phospho‐mimicking hCLU mutant CLU_S396D_ was overexpressed in mouse brains, the expression level of this mutant in the brain was 3–4 folds higher than that of the wild‐type hCLU overexpressed in the brain, but in the sera, the level of the mutant was only 20%–30% of the wild‐type hCLU (Figure 4D). Hence, even if CLU is phosphorylated in the brain, the phospho‐CLU was inefficiently secreted into the blood. Therefore, brain‐derived phospho‐CLU, if present, may not significantly contribute to phospho‐CLU in the serum.

### 3.5 Brain‐Derived CLU Is Not Phosphorylated During Transcytosis in AD‐Like Pathology

**FIGURE 5:**
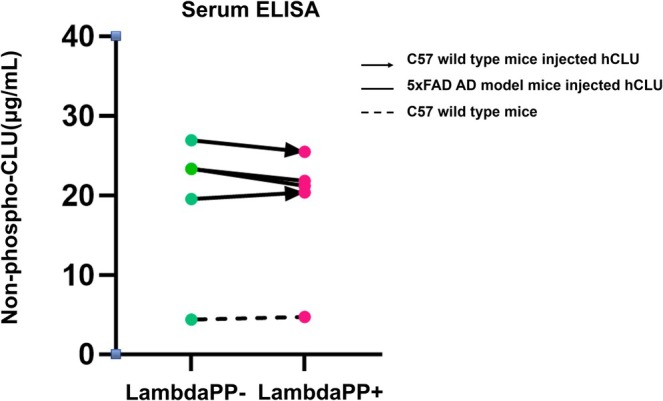
Brain‐derived CLU is unlikely to be phosphorylated during its excretion into the bloodstream. Wild‐type hCLU was overexpressed in the brains of wild‐type C57 mice and 5XFAD AD model mice. Serum hCLU levels, treated with (LambdaPP+) or without λ‐protein phosphatase (LambdaPP−), were quantified using ELISA with the 3D3F10 antibody. Control AAV injected WT mice: *n* = 1, WT mice overexpressing hCLU: *n* = 2, 5XFAD mice overexpressing hCLU: *n* = 2.

Next, we wondered if brain‐produced CLU could be phosphorylated at the three sites when it is transcytosed from the brain to the circulation, especially in the context of AD. AAV expressing wild‐type hCLU was ICV injected into C57 wild type mice and 5‐month‐old 5× FAD AD model mice. At this age, 5× FAD mice show impaired cognition and neuritic plaques are abundant in the brain. However, ELISA using 3D3F10‐coated plates revealed no increase in the readout when the sera from these mice were treated with λ‐protein phosphatase (Figure 5). Therefore, under our experimental condition with a limited number of mice, no CLU phosphorylation during transcytosis was detected.

## 4 Discussion

In recent years, blood‐based biomarkers—such as the amyloid beta [36, 37, 38], phosphorylated tau (p‐tau) [39, 40, 41], glial fibrillary acidic protein (GFAP) [42, 43]—have demonstrated potential for clinical applications in predicting, diagnosing, and monitoring the progression of Alzheimer's disease. However, due to the extremely low concentrations of these biomarkers in the blood, their detection requires highly sensitive equipment and reagents, thereby limiting their widespread clinical application [10, 44].

In this study, we developed the first monoclonal antibody (3D3F10) specifically recognizing non‐phosphorylated clusterin (CLU) at residues Thr393, Ser394, and Ser396. Using this novel tool in a pilot cohort, we demonstrated that serum non‐phospho‐CLU displays a potential to distinguish dementia patients from non‐demented controls (AUC = 0.897, sensitivity 81.8%, specificity 95.5%), while showing no discrimination for Parkinson's disease. Unexpectedly, the change in non‐phospho‐CLU was opposite to our initial hypothesis. It was decreased instead of being increased in dementia. Our subsequent experimental results using 3D3F10 indicated that the increased phospho‐CLU in serum (as deduced from the reduction of non‐phospho‐CLU) was unlikely derived from the brain. It should be noted that a reduction in 3D3F10 signal does not directly demonstrate an increase in phosphorylated CLU, as other PTMs (e.g., glycosylation) or conformational changes at this epitope could produce a similar result. Throughout this study, “phospho‐CLU” is used as shorthand for “CLU modified at the 3D3F10 epitope,” but definitive identification of the responsible modification(s) will require targeted mass spectrometry.

The increase of CLU in brain parenchyma [16, 17, 18, 19, 20], CSF [21, 22, 23], and plasma [19, 24, 25, 26, 27] of AD patients has been well documented. However, due to the large inter‐individual variability in total plasma CLU, its clinical utility as a biomarker has not been developed [45]. We previously reported that removing lipoprotein‐bound CLU from serum improved diagnostic efficacy [15], but this method is not efficient enough for wide clinical application. The 3D3F10 antibody, which directly detects the non‐phosphorylated form of CLU, offers a more convenient assay and achieved even higher efficacy than the lipoprotein‐removal approach. Whether or not CLU could be used for diagnosis of dementia has not been investigated. Our data suggest that non‐phospho‐CLU (or CLU not modified otherwise at the 3D3F10 epitope) detected by 3D3F10 may facilitate the translation of CLU‐based testing into clinical practice for dementia diagnosis. It should be noted that phosphorylation at Thr393, Ser394, and Ser396 is the known post‐translational modification (PTM) within the 3D3F10 epitope, but other modifications such as glycosylation or sulfation might also occur at these residues in diseases. For instance, CLU glycosylation at N64 is increased in the CSF of AD patients [45], and N64 glycosylation in plasma CLU is decreased in individuals with high hippocampal atrophy [46]. Although there is currently no report indicating PTMs other than phosphorylation within 3D3F10 epitope, if glycosylation or other PTMs occur at the three phosphorylation sites, they might interfere with 3D3F10 binding. Therefore, future studies are needed to determine whether phosphorylation is the sole modification of 3D3F10 reactivity in human serum and how other epitope‐modifying PTMs correlate with dementia.

Mechanistically, we found that phosphorylation at Thr393, Ser394, and/or Ser396 alters the secondary structure of the epitope of 3D3F10. Circular dichroism analysis revealed that in vitro phosphorylation induced a conformational change in the synthetic epitope. This structural rearrangement likely reduces the accessibility of the 3D3F10 epitope, providing a molecular basis for the antibody's specificity for the non‐phosphorylated form. Consistently, 3D3F10 did not react with CLU in Western blot where proteins are denatured by SDS. Moreover, because protein conformation critically influences interactions with binding partners, clearance rates, and chaperone activity, phosphorylation‐induced structural changes in CLU may have functional consequences beyond epitope masking—potentially affecting CLU's role in lipid binding, Aβ aggregation, or receptor‐mediated signaling. These possibilities warrant further investigation.

Although we initially hypothesized that brain‐derived CLU would elevate serum non‐phospho‐CLU after entering the circulation, our findings contradicted this prediction. Non‐phospho‐CLU was decreased in dementia patients, suggesting that the fraction of CLU modified at the 3D3F10 epitope is increased in disease. It is also possible that the 3D3F10 epitope is structurally changed in dementia. We therefore investigated whether the phosphorylated (or otherwise modified) CLU originates from the brain, because although phospho‐CLU has not been detected in brain parenchyma, since CLU is a secretory protein, it might be phosphorylated soon after synthesis and secreted into interstitial fluid. However, using 3D3F10‐based ELISA, we found little or no phospho‐CLU in CSF from AD patients. Moreover, in mice artificially overexpressing human CLU specifically in the brain, serum levels of human CLU did not show enhanced 3D3F10 reactivity after dephosphorylation treatment, arguing against a brain origin for the modified circulating CLU. Finally, phospho‐mimicking mutants of CLU were far less efficiently secreted from the brain into the blood than wild‐type CLU. Together, these data suggested that the increased phospho‐CLU (or CLU with 3D3F10 modified by other PTMs) in dementia serum is predominantly produced in peripheral tissues.

A small amount of CLU phosphorylation was detected in the CSF of some AD patients, but this does not necessarily imply phosphorylation in brains. Approximately 48% of AD patients have moderate‐to‐severe cerebral amyloid angiopathy (CAA) [47], which can cause microhemorrhages and compromise blood–brain barrier integrity [48, 49]. It has been shown that the peripheral immune system plays a significant role in the progression of AD and dementia. The disruption of the blood–brain barrier (BBB) enables peripheral immune cells to infiltrate the central nervous system (CNS) [50, 51]. The peripheral phospho‐CLU might enter the CSF through the leaky blood–brain barrier. Alternatively, CLU could be phosphorylated within brain endothelial cells during transcytosis from blood to brain. However, our mouse model data argue against significant brain‐derived phosphorylation of CLU in the blood. Hence, the modified CLU at the 3D3F10 epitope appears to be largely peripheral in origin.

CLU is widely expressed in various organs, and several peripheral conditions closely associated with dementia—such as cardiovascular disease, hyperlipidemia, and diabetes—are known to upregulate total blood CLU [52, 53, 54]. Cardiovascular disease and hyperlipidemia may impair CLU metabolism, potentially reducing the non‐phosphorylated population. For example, high glucose has been reported to induce serine phosphorylation of CLU in hepatocytes [29]. Thus, the elevated serum phospho‐CLU (inferred from the decrease in non‐phospho‐CLU, and assuming no other epitope modifications) in dementia patients might reflect peripheral comorbidities rather than brain pathology. The structural changes induced by phosphorylation, as observed in our circular dichroism experiments, may also affect CLU's half‐life or tissue distribution, further contributing to the altered serum levels. Given our small sample sizes and the baseline conditions such as diabetes, cardiovascular disease, and hyperlipidemia were not available for this cohort, the potential of non‐phospho‐CLU as a diagnostic marker for dementia should be interpreted with caution.

The functional consequences of CLU phosphorylation at these sites remain largely unknown. Phosphorylation could alter CLU's structure and biological activities, including its excretion from the brain (as suggested by our mutant CLU experiments in Figure 4D). Notably, peripherally administered recombinant CLU (which is non‐phosphorylated) suppresses neuroinflammation and AD pathology in mouse models, suggesting that non‐phosphorylated CLU in blood may play a protective role even if it could not enter brain parenchyma [31]. If peripheral CLU becomes phosphorylated in dementia—and if this phosphorylation induces the conformational changes we observed—this protective function might be impaired. Investigating whether phosphorylation of peripheral CLU contributes to dementia pathogenesis could provide new insights into how peripheral changes influence brain function.

Several practical and operational issues may emerge during the development and validation of 3D3F10: (1) Our observations were made in serum samples, and it should be cautious when plasma samples are tested. In our own data and some other reports, CLU levels in the sera of AD patients and healthy controls are similar [15, 29], therefore, a lower percentage of non‐phospho‐CLU may translate into a lower level of non‐phospho‐CLU. However, CLU in plasma of AD is significantly higher by statistics [19], hence, a lower percentage of non‐phospho‐CLU does not necessarily result in a lower level. (2) The antibody failed to detect CLU by Western blot under denaturing conditions. This limitation, which we attribute to phosphorylation‐induced conformational changes disrupting the epitope, necessitates the use of native assays without disrupting structures. (3) While the antibody demonstrates high specificity for non‐phosphorylated CLU in controlled experimental settings, its performance in clinical samples may be influenced by pre‐analytical factors. Sample storage time, freeze–thaw cycles, and handling conditions could potentially affect the stability of the phosphorylation state or the epitope itself. (4) The possibility of other post‐translational modifications within the 3D3F10 epitope (such as glycosylation or sulfation) cannot be excluded. While currently no such modifications have been reported at these specific residues, their potential presence in disease states could interfere with antibody binding and should be investigated in future studies.

This proof‐of‐concept study has several limitations. The sample size is modest (*n* = 22 per group), and the diagnostic accuracy needs validation in larger, independent cohorts that include diverse dementia subtypes (e.g., AD, vascular dementia) and mild cognitive impairment. Additionally, while there is currently no PTM other than phosphorylation of CLU at the three sites, the precise nature of the PTM(s) masking the 3D3F10 epitope in serum remains to be fully characterized—it could be phosphorylation, glycosylation, or other modifications. Future studies employing mass spectrometry are needed to resolve this question and to examine whether these modifications correlate with specific clinical features or comorbidities. Furthermore, while our structural data provide initial evidence for phosphorylation‐induced conformational changes, high‐resolution structural studies would be valuable to precisely map the epitope and the structural basis of 3D3F10 specificity. Despite these limitations, this study establishes a first‐in‐class antibody that not only holds diagnostic promise but also provides a new conceptual framework for understanding the peripheral origins of circulating CLU modifications in dementia.

In summary, we have developed and validated the first‐in‐class antibody specifically recognizing non‐phosphorylated clusterin. Using this novel tool in a pilot cohort, we demonstrate that serum non‐phospho‐CLU has the potential of distinguishing dementia from non‐demented controls. Although disease specificity should be further examined, non‐phospho‐CLU at least did not differentiate PD. We also provide the first experimental evidence that circulating phospho‐CLU does not originate from the brain, but more likely from peripheral tissues. This proof‐of‐concept study establishes non‐phospho‐CLU as a promising candidate biomarker and opens new avenues for investigating peripheral contributions to dementia pathophysiology. Larger‐scale validation is warranted to translate these findings into clinical applications.

## Author Contributions

Z.W. designed this study. H.L., Y.L., W.L., Q.R., and X.L. performed the experiments. L.W. and Y.C. provided clinical samples. C.H. performed statistical analysis. H.L., Y.C., and Z.W. wrote this manuscript.

## Funding

This work was supported by the MOST|National Natural Science Foundation of China (NSFC) (81870832).

## Ethics Statement

The use of human samples was approved by the Ethics Committees of Xuanwu Hospital of Capital Medical University on October 12, 2020 (approval number: [2020]104) and on March 20, 2022 (approval number: [2022] 047), and conducted in accordance with the ethical principles outlined in the Declaration of Helsinki. Written informed consent was obtained from all the participants or their legal guardians. Patients and healthy controls were recruited by co‐authors who are clinical physicians, and the use of blood samples is covered by the ethical approvals. Ages and genders of the subjects recruited to this work were reported before. Animal experiments were carried out in accordance with the guidelines for the Care and Use of Laboratory Animals from Xuanwu Hospital, Capital Medical University (approval number: 20200108).

## Consent

The authors have nothing to report.

## Conflicts of Interest

The authors declare no conflicts of interest.

## Supporting information


**Table S1:** Demographic statistics of dementia and PD patients.

**Table S2:** CVs of ELISA for each sample.

## Data Availability

The authors have nothing to report.
